# Effectiveness of near-UVA in SARS-CoV-2 inactivation

**DOI:** 10.1017/S0950268823000560

**Published:** 2023-04-27

**Authors:** Eleonora Frilli, Davide Amodeo, Gabriele Cevenini, Nicola Nante, Gabriele Messina

**Affiliations:** 1 Post-Graduate School of Public Health, University of Siena, Siena, Italy; 2 Hygiene and Preventive Medicine, University of Siena, Siena, Italy; 3 Department of Medical Biotechnologies, University of Siena, Siena, Italy

**Keywords:** COVID-19, virus infection, SARS-CoV-2, Cell Colture, near-UVA, UVC, Disinfection, Hygiene, Prevention

## Abstract

This experimental study aimed to determine the activity of a near-UVA (405 nm) LED ceiling system against the SARS-CoV-2 virus. The ceiling system comprised 17 near-UVA LED lights with a radiant power of 1.1 W/each centred at 405 nm wavelength. A 96-multiwell plate, fixed to a wooden base, was inoculated with suspensions of VERO E6 cell cultures infected with SARS-CoV-2 virus and irradiated at a distance of 40 cm with a dose of 20.2 J/cm^2^ for 120 min. The collected suspensions were transferred to VERO cell culture plates and incubated for 3 days. The maximum measurable log reduction obtained, starting from a concentration of 10^7.2^ TCID50/mL, was 3.0 log_10_ and indicated inhibition of SARS-CoV-2 replication by the near-UVA LED ceiling system. Near-UVA light at a 405-nm wavelength is emerging as a potential alternative treatment for localised infections and environmental decontamination because it is far less harmful to living organisms’ cells than UV-C irradiation.

## Introduction

In December 2019, the first cases of COVID-19 disease were reported in the Hubei province, China, before becoming a global pandemic [1]. The pathogen was identified as a beta-Coronavirus and designated as SARS-CoV-2 [2], which up to February 2023 had caused over 674 million infections with more than 6 million deaths worldwide [3]. Coronaviruses are mainly transmitted via respiratory droplets, but due to their prolonged viability in air and on inanimate surfaces, the virus particles may retain infectivity for several hours [4].

Irradiation using ultraviolet (UV) light is an established disinfection strategy for surfaces, air and water in hospitals to reduce the risk of infection [5]. It has been shown that coronaviruses are generally susceptible to UV radiation [6] and in particular to UVC [7]. UVC (wavelength 200–280 nm) has a greater germicidal effect than UVA (315–380 nm) and UVB (280–315 nm), as it acts directly on nucleic acid bases causing structural damage through photodimerisation. However, lately, near-UVA (405 nm) has emerged as a potential alternative treatment for localised infections and environmental decontamination [8]. UVC and near-UVA irradiation are the most widely used light disinfection methods, with the latter being less detrimental to host cells [9]. Near-UVA, particularly of the 405 nm wavelength, exerts its antimicrobial effects through excitation of endogenous intracellular light receptors such as porphyrins and flavins [10] which then undergo an energy transfer process that leads to the generation of reactive oxygen species (ROS) resulting in lipid peroxidation, DNA damage, cell wall damage, and cellular apoptosis of microbial cells [11]. Several studies have explored the effects of near-UVA against bacterial and fungal pathogens [12].

However, the ability of near-UVA to inactivate viruses, in particular, the SARS-CoV-2, which lack the porphyrin rings targets have received relatively little attention [13], with the possible exception of the work of Vatter et al. [14] investigating the photoinactivation of a Coronavirus surrogate, phi6, using light at a 405 nm wavelength.

The aim of this study is to assess the virucidal activity of a 405 nm LED ceiling system directly against SARS-CoV-2 *in vitro* through its impact on the viability of a SARS-CoV-2 strain in a Tissue Culture Infective Dose 50% (TCID_50%_) model.

## Methods

An experimental pre–post design study was undertaken, to assess the virucidal activity of a 405 nm LED ceiling system against SARS-CoV-2. The experiment took place partly in the Department of Molecular and Development Medicine at the University of Siena, for the experimental setting building stage, and partly in a Biosafety Level 3 Laboratory (BLS3) of Toscana Life Sciences (the technology park for the Southern Tuscany region, Italy), for *in vitro* tests.

### 405 nm light system and setup

**Figure 1.**
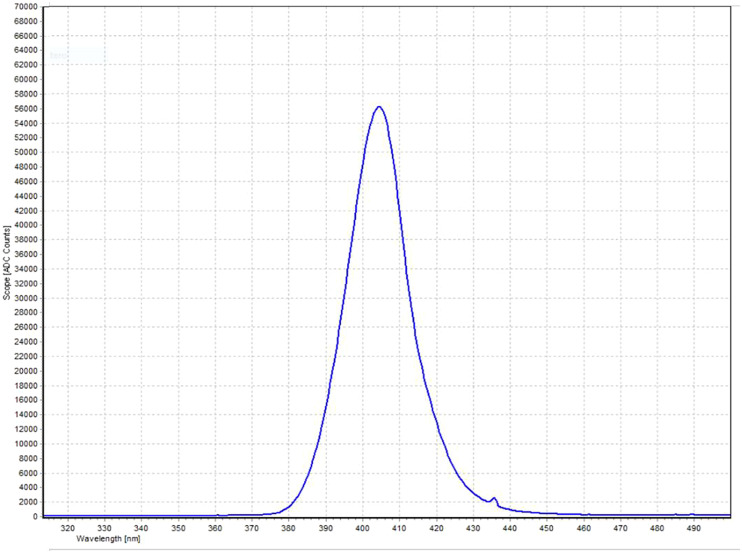
Spectral behaviour as a function of wavelength (nm) of the near-UVA LEDs Luminus SST-10-UV (blue line).

The near-UV CEILING LAMP System at 405 nm (Figure 1) comprised 17 blue LED Luminus SST-10-UV lights (Luminus, Sunnyvale, CA, USA) positioned on a squared area of a printed circuit board (PCB); the distance between each LED was 6.5 cm. The LAMP System was supported by a wooden scaffold, fixed to a wooden base. The wooden structure, built for the experiment, consisted of four vertical supports, each 40 cm high, and a 36 cm x 36 cm wide wooden base the same size as the ceiling lamp used for the test. An outline of a multiwell plate, consisting of 24 flat bottom wells, was drawn in the central position of the base to facilitate correct positioning during the test.

**Figure 2.**
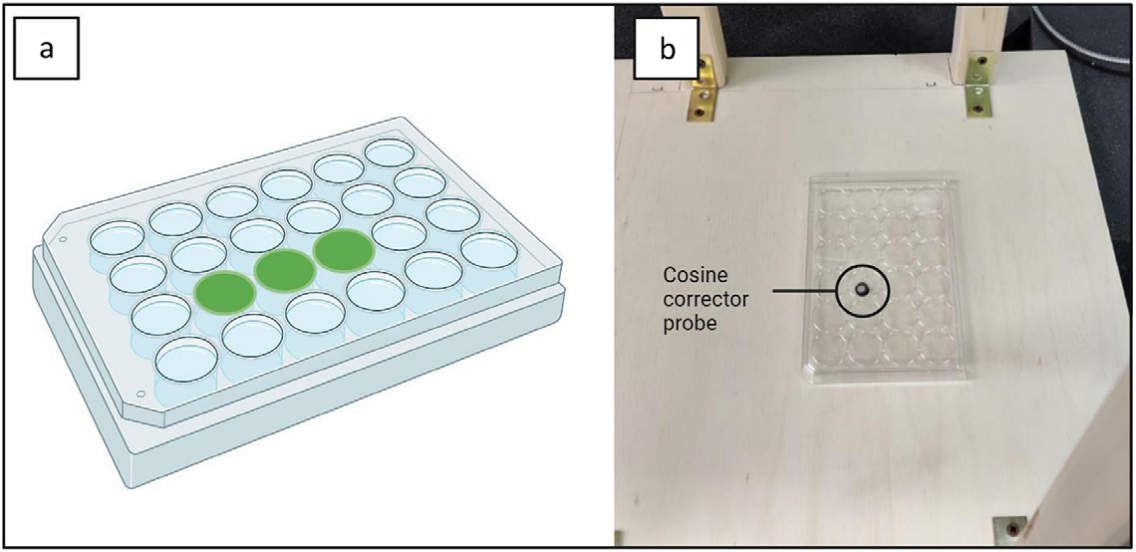
(a) Example of the 24 multiwell plate and selected wells used for the test (green spots C2 on the left, C3 in the centre, and C4 on the right). (b) Photometric measurements were carried out with the cosine corrector probe of the Avantes spectrophotometer to detect the irradiance values present in each of the three wells of the plate.

Irradiance measurements were made using a spectrometer Avantes ULS2048CL-EVO (Avantes, Apeldoorn, the Netherlands) on the multiwell plate in the central position with plate cover on, as shown in Figure 2. Light distribution was not uniform over the whole plate and therefore, only the three central wells were used in each multiwell plate to minimise variation (maximum deviation in measured irradiance = 0.1 mW/cm^2^). Irradiance values and exposure times allowed calculation of the energy doses.

### Cell culture and viruses

Vero E6 cells of the African green monkey kidney line (American Type Culture Collection [ATCC] #CRL-1586) were cultured in Dulbecco’s Modified Eagle’s Medium (DMEM) – high glucose (Euroclone, Pero, Italy) supplemented with 2 mM l-glutamine (Lonza, Milan, Italy), penicillin (100 U/mL) – streptomycin (100 μg/mL) (Lonza, Milan, Italy), and 10% foetal bovine serum (FBS) (Euroclone, Pero, Italy). Cell cultures were infected with the wild-type SARS CoV-2 2019 virus (2019-nCoV strain 2019-nCov/Italy-INMI1) (EVAg, Istituto Spallanzani, Rome, Italy). For virus propagation, monolayers of subconfluent Vero E6 cells were prepared in T175 flasks containing high-glucose DMEM medium.

### Experimental method

**Figure 3.**
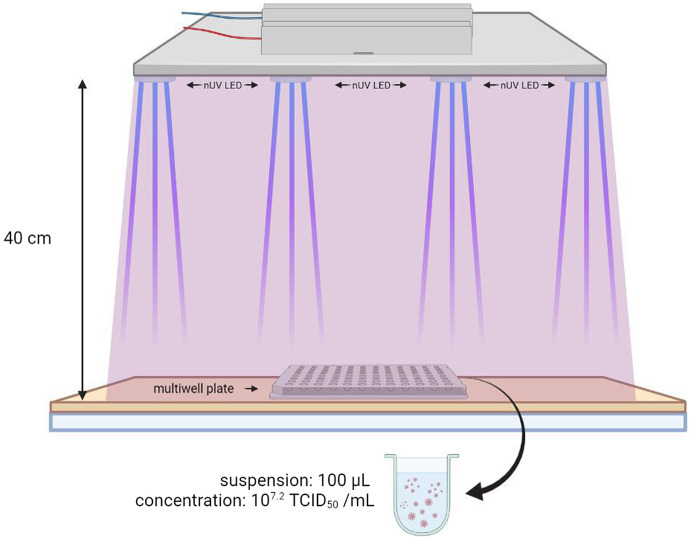
Experiment setting designed for the tests with the 405 nm LED ceiling system.

One hundred microlitres of viral suspension (10^7.2^ TCID_50_/mL), were inoculated in each plate well in phosphate-buffered saline (PBS; Oxoid, Basingstoke, UK). The plate was irradiated according to the protocol at a distance of 40 cm, for 30 and 120 min (Figure 3). For each exposure time, tests were carried out in triplicate. To do this, three aliquots of the viral suspension were delivered in left, central, and right positions of the plate (and exposed to the action of nUV with protocol timing. Three other aliquots were inoculated in another multiwell plate (shielded from light and not exposed to near-UVA) which served as a positive control to determine viral titre after recovery.

Following the exposure, the viral suspension was transferred to a 96-multiwell plate and 20 μL of suspension supplemented with 180 μL of high-glucose DMEM medium were transferred to each well of the first column and decimal dilutions were made. The suspensions were then inoculated into a new 96-multiwell plate into which the VERO E6 cell cultures were fixed, and then incubated for 3 days at 37 °C ± 2 °C at 5% CO_2_ in a humidified atmosphere. Each plate was examined with an optical microscope to assess the exhibited cytopathic effect (CPE). Viral titres were determined using a TCID_50_ assay. Experiments were repeated in triplicate, carried out independently from each other, and under the same measurement conditions. Untreated control cells were used to validate the test. The virus titre was calculated using Spearman–Karber’s formula [15] and defined as the reciprocal of the highest viral dilution leading to at least 50% CPE in inoculated wells.

## Results

The irradiance value measured on the three selected central wells of the multiwell plate was 2.8 mW/cm^2^ and the amount of total energy dose received in 30 and 120 min during exposure to near-UVA was equal to 5.05 and 20.20 J/cm^2^, respectively.

The results in Table 1 show that i) after an irradiation time of 30 min, the maximum measurable log reduction TCID_50%_ was equal to 1.50; and ii) at 120 min, the maximum TCID_50%_ was equal to 3.00. The percentage reductions and relative 95% confidence intervals were 96.17% [94.37%–97.39%] and 99.85% [99.78%–99.90%], at irradiation times of 30 and 120 min, respectively.

**Table 1.** Results of the 405 nm LED ceiling system at 40 cm from the multiwell plate after 30 and 120 min of near-UVA exposition

Distance from light source	Contact time	Multiwell plate position (co-ordinates)	SARS-CoV-2 Log suspension TCID_50%_ (untreated virus suspension)	SARS-CoV-2 Log suspension TCID_50%_ (UV-treated virus suspension)	Log reduction TCID_50%_
40 cm	30 min	Left position (C2)	5.00	3.75	1.25
		Central position (C3)	4.75	3.25	1.50
		Right position (C4)	5.00	3.50	1.50
	120 min	Left position	4.25	1.50	2.75
		Central position	4.25	1.50	2.75
		Right position	4.50	1.50	3.00

TCID, median tissue culture infectious dose.

## Discussion

Since the beginning of the COVID-19 pandemic, several studies have explored different disinfection strategies against coronaviruses and some have involved light disinfection methods. Nevertheless, only a few studies have addressed the activity of near-UVA against viruses, particularly SARS-CoV-2 [13, 14].

Here, we have shown that a 405-nm LED ceiling system (Figure S1) is effective for the inactivation of SARS-CoV-2, *in vitro.* It was found that an energy dose of approximately 5 J/cm^2^, delivered a reduction of over 96% (1–2 log_10_), while a dose four times higher (about 20 J/cm^2^) almost reached a reduction of 99.9% (3 log_10_). These results are quite different from those observed by Tomb et al. [16] who reported only a 0.3 log reduction with a dose of 306.2 J/cm^2^ in inactivation studies of Streptomyces phage phiC31 in phosphate-buffered saline. This could be due to the fact that phiC31, in contrast to SARS-CoV-2, is a non-enveloped dsDNA virus. Similarly, Vatter et al. [14] observed a 1 log reduction with a dose of 430 J/cm^2^ for a Coronavirus surrogate in PBS, but used a *Pseudomonas syringae* cell culture instead of our VERO E6 cells and likely also a different initial virus concentration. It is also noteworthy that the viral inoculum in our experiments was suspended in PBS rather than a nutrient solution to exclude the presence of external photosensitisers.

Since near-UVA requires longer exposure times or a higher radiant energy than UV-C for inactivating viruses, SARS-CoV-2 included [17], it is less energy efficient when compared with other available pulsed-light technologies [18] particularly at the UV-C wavelength of mercury lamps (254 nm) [19]. Although near-UVA can represent a feasible alternative because of its safety [20], it is important to note some possible negative effects of lights of a wavelength > 405 nm. The blue light band (415–455 nm) is harmful to retinal pigment epithelial cells [21], while wavelengths of 480–490 nm appear to influence physiological responses, such as mood, pupillary constriction [22], and circadian rhythm due to maximum suppression of melatonin at 484 nm [23]. Nevertheless, it has been shown that mammalian keratinocytes and osteoblasts were more resistant than bacteria on exposure to bactericidal levels of 405 nm light, with no loss of cell viability [24].

Furthermore, prolonged exposure of materials, such as plastic, paint, and other coating substances, to a 405 nm light induces less photo-degradation than UV-C irradiation (254–260 nm). Therefore, near-UVA light around 405-nm appears to be safe for continuous use even in the presence of people, with a quantity of light energy as a suitable trade-off between avoiding photobiological risks and ensuring a satisfactory disinfection level [25]. The distance at which the tests were carried out was limited to only 40 cm to safely manage the tests under the hood.

A limitation of this study is that the inactivation of SARS-CoV-2 was evaluated in a liquid media and not in aerosolised droplets, considering that they are the main infection route, so further studies are indicated as the near-UVA effects may depend on the substrate considered. Despite this, the ability to inactivate the virus on objects where the virus should settle or in environments makes this technology potentially useful to reduce the risk of infection.

Another important limit to this technology is linked to its photobiological risk. It is important to note that the wavelengths used encompassed a wider range, with peak inactivation at 405 nm, but potentially hazardous photobiological effects on skin and eyes [26] linked to wavelengths under 400 nm cannot be discounted and guidelines for the photobiological safety assessment of these radiation wavelengths have been published [27]. For this reason, it is necessary to manage aspects of safety with precautionary technical expedients by positioning the light sources at appropriate distances, and in compliance with international standards.

In conclusion, this study has demonstrated that near-UVA light is effective for the inactivation of SARS-CoV-2. Viability of the virus in a non-nutrient medium was reduced by 96.17 and 99.8% after exposures to the light source for 30 and 120 min, respectively. Application of this technology could reduce the risk of infection and increase the level of environmental hygiene, especially when associated with other physical or chemical disinfection methods.

## Data Availability

The data that support the findings of this study are available from the corresponding author, G.M., upon reasonable request.

## Supplementary material

For supplementary material accompanying this paper visit https://doi.org/10.1017/S0950268823000560. click here to view supplementary material
