# Supplementary figures and images for: Effectiveness of near-UVA in SARS-CoV-2 inactivation
Source: Epidemiol Infect. 2023 Apr 27;151:e76. doi: 10.1017/S0950268823000560 (PMC10203533; doi:10.1017/S0950268823000560)

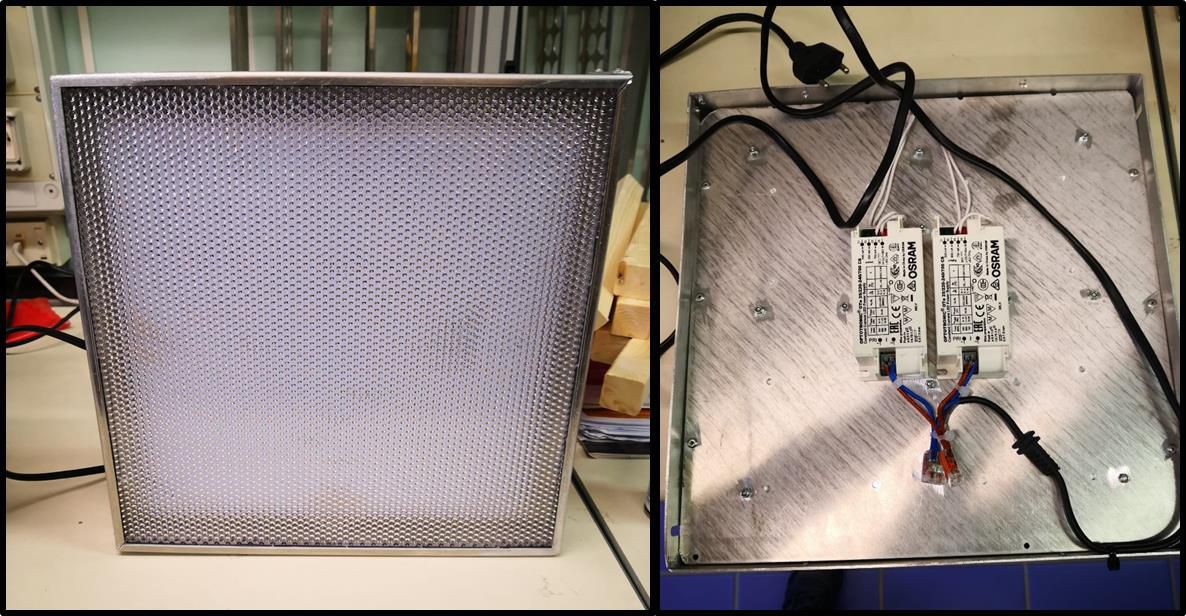

Supplement: Supplementary file 1 [file S0950268823000560sup001.jpg]
